# Templated fabrication of hierarchically porous metal–organic frameworks and simulation of crystal growth†

**DOI:** 10.1039/c8na00262b

**Published:** 2018-12-12

**Authors:** Chongxiong Duan, Hang Zhang, Minhui Yang, Feier Li, Yi Yu, Jing Xiao, Hongxia Xi

**Affiliations:** School of Chemistry and Chemical Engineering, South China University of Technology Guangzhou 510640 PR China cehxxi@scut.edu.cn; Guangdong Provincial Key Laboratory of Atmospheric Environment and Pollution Control, South China University of Technology, Guangzhou Higher Education Mega Centre Guangzhou 510006 PR China

## Abstract

Hierarchically porous metal–organic frameworks (MOFs) have recently emerged as a novel crystalline hybrid material with tunable porosity. Many efforts have been made to develop hierarchically porous MOFs, yet their low-energy fabrication remains a challenge and the underlying mechanism is still unknown. In this study, the rapid fabrication of two hierarchically porous MOFs (Cu-BTC and ZIF-8) was carried out at room temperature and ambient pressure for 10 min using a novel surfactant as the template in a (Cu, Zn) hydroxy double salt (HDS) solution, where the (Cu, Zn) HDS accelerated the nucleation of crystals and the anionic surfactants served as templates to fabricate mesopores and macropores. The growth mechanism of hierarchically porous MOFs was analyzed *via* mesodynamics (MesoDyn) simulation, and then the synthetic mechanism of hierarchically porous MOFs at the molecular level was obtained. The as-synthesized hierarchically porous Cu-BTC showed a high uptake capacity of 646 mg g^−1^, which is about 25% higher as compared with microporous Cu-BTC (516 mg g^−1^) for the capture of toluene. This study provides a theoretical basis for the large-scale fabrication of hierarchically porous MOFs and offers a reference for the understanding of their synthetic mechanism.

## Abstract 2

Hierarchically porous MOF materials were rapidly synthesized in a (Cu, Zn) hydroxy double salt (HDS) solution using a novel surfactant as template at room temperature and ambient pressure, and their growth mechanism was disclosed *via* mesodynamics (MesoDyn) simulation.

## Graphical abstract

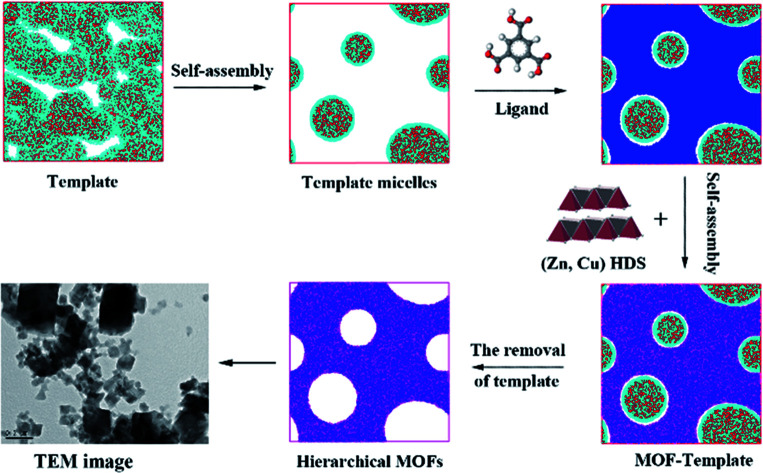

## Introduction

Metal–organic frameworks (MOFs), consisting of organic ligands and metal ions (or clusters), have drawn intense interest as a novel synthetic porous material.^1,2^ In comparison to traditional porous solids such as activated carbon, mesoporous silica, and zeolites, MOFs have diverse topology structures, high surface area, ultrahigh porosity, and multiple tunable functions.^3–5^ Therefore, it can be used in a wide range of applications, such as adsorption/separation,^6,7^ photoelectronics,^8,9^ energy conversion,^10,11^ drug delivery,^12^ chemical sensing,^13^ and catalysis.^14^ Actually, the size and shape of pores within MOFs are one of decisive parameters for the above-mentioned applications, since the guest species accessing or leaving the internal active sites of MOFs is a prerequisite for the proceeding of reaction.^15,16^ However, the pores of most available MOFs to date have belonged to a pure microporous regime (pore size < 2 nm),^17^ which would impede the diffusion rate and restrict the accessibility of guest molecules toward the active sites within the internal space of MOFs, especially for biomacromolecules.^18^ Therefore, it is of great importance to develop an effective and reliable strategy to transform microporous MOFs into their hierarchical porous counterparts with micropores and mesopores.

To overcome the limitation of pore size, hierarchically porous MOFs with micropores and large pores (mesopores and macropores) have become a subject of great interest due to their advantageous integration of micro- and mesoporosity.^19^ Specifically, micropores would conduce to the high surface area and abundant active sites, while the mesopores and macropores across the microporous matrix would facilitate the diffusion and transport of large guest molecules to approach or leave the active sites inside the micropores.^20,21^ To date, tremendous efforts have been devoted to preparing hierarchically porous MOFs,^22,23^ and two major synthetic approaches have been developed.^24^ One available approach, also known as the ligand extension method, is to form intrinsic mesopores in MOFs through either increasing the length of organic ligands or applying large organic scaffolds.^25^ Unfortunately, the largest mesopore size of the MOFs synthesized with this method is usually smaller than 10 nm.^26^ Another approach is the supramolecular template strategy, which generates mesopores and macropores in MOF crystals by using a surfactant as the template.^24,27^ However, there are issues related to long reaction times, high temperatures and pressures, and complicated synthetic procedures and equipment.^28–30^ Therefore, it is highly desirable but remains a great challenge to develop a simple, reliable, and energy-saving method to rapidly synthesize hierarchically porous MOFs under mild conditions.

To date, many researchers have proposed various synthetic mechanisms of hierarchically porous MOFs; however, most of them are based on the experimental results, and only a few have been reported based on the investigation of hierarchically porous MOFs using a computer-assisted method at molecular and atomic levels.^31^ As a matter of fact, computational simulation can shed considerably valuable light on the mechanism study to investigate the synthetic mechanism of porous materials at the molecular level.^32,33^ For example, the mesoscopic dynamics (MesoDyn) simulation method, which is based on dynamic mean-field density functional theory (DFT),^34^ is a powerful analytical approach.^35^ It has recently been extensively applied in investigating the synthetic mechanism of porous materials. For instance, Zhang *et al.*^36^ had utilized the MesoDyn simulation method to simulate the microphase behavior of a triblock copolymer (P65) in aqueous solution. Chen *et al.*^37^ had employed the MesoDyn method to examine the mesophase formation in mesoporous zeolites (SBA-15). In addition, Mu *et al.*^38^ had simulated the phase morphology and self-assembly structure of a symmetric amphiphilic copolymer in thin films using the MesoDyn method. However, to the best of our knowledge, few studies have been reported on the use of MesoDyn simulation to examine the phase behavior of templates and to understand the synthetic mechanism of hierarchically porous MOFs.^39^

In this study, the aforementioned issues were addressed simultaneously. Two hierarchically porous MOFs (Cu-BTC and ZIF-8) were rapidly synthesized in a (Cu, Zn) hydroxy double salt (HDS) solution using a novel surfactant (*N*,*N*-dimethyloctylamine, DMOA) as the template at room temperature and ambient pressure. During the synthesis, the DMOA surfactant was used to fabricate mesopores and macropores while HDS played a key role in accelerating the nucleation of crystallization. The synthesis time can be shortened dramatically to 10 min only. Furthermore, the as-prepared hierarchically porous Cu-BTC exhibited hierarchically porous structures with micro-, meso-, and macropores as well as excellent thermal stability. Moreover, the porosity of H-MOFs was tuned rationally by adjusting the amount and type of surfactant employed. Other than DMOA, it is worth noting that other surfactants such as *N*,*N*-dimethyldodecylamine (DMDA) can be used as templates for the rapid preparation of hierarchically porous MOFs. What is more, this study investigated the phase behavior of the template and the synthetic mechanism of hierarchically porous MOFs using the MesoDyn simulation method. In comparison with primitive microporous MOFs, the attained hierarchically porous MOFs exhibited a higher toluene uptake capacity due to the presence of mesopores and macropores. The strategy developed in this work enables us to achieve rapid synthesis of hierarchically porous MOFs at room temperature and pressure.

## Materials and methods

### Rapid room-temperature synthesis of hierarchically porous Cu-BTC

In a typical synthesis,^40,41^ firstly, 3.6 mmol of zinc oxide (ZnO) powder was dispersed in 8 mL deionized water and 16 mL *N*,*N*-dimethylmethanamide (DMF) to form a nanoslurry through using sonication for 15 min (solution A). Secondly, 7.2 mmol of copper nitrate trihydrate (Cu(NO_3_)_2_·3H_2_O) was dispersed in 18 mL of deionized water as solution B, then the two solutions (solution A and solution B) were mixed under fast stirring to form a (Zn, Cu) hydroxy double salt (HDS) (solution C).^42^ After that, stirring was continued for 15 min and then sonication for 10 min at room temperature. A micelle solution was prepared by adding 2.5 mmol of *N*,*N*-dimethyloctylamine (DMOA) and 4 mmol of 1,3,5-benzenetricarboxylic acid (H_3_BTC) to 15 mL of ethanol (solution D). After stirring for 30 min, the two solutions (solution C and solution D) were combined and continuously stirred for 10 min. Subsequently, a glaucous precipitate was collected by filtration and immersed in ethanol solution 4 times at 60 °C for 48 h, and then dried overnight in an oven at 120 °C. The resultant product is denoted as H-Cu-BTC. Similarly, hierarchically porous Cu-BTC samples synthesized with various molar concentrations of DMOA are denoted as H-Cu-BTC_1 (DMOA = 1.0 mmol) and H-Cu-BTC_5 (DMOA = 5.0 mmol), respectively.

## Materials characterization

Powder X-ray diffraction (XRD) patterns of hierarchically porous MOFs were analyzed with a diffractometer system (D8 ADVANCE, Bruker AXS) with a Cu sealed tube (40 kV, 40 mA, wavelength *λ* = 0.15418 nm) Fourier transform infrared (FTIR) spectra of samples were measured with an FTIR spectrometer (Vector 33, Bruker Corporation) with a resolution of 4 cm^−1^. Nitrogen (N_2_) adsorption–desorption isotherms were recorded using an ASAP 2020 and 2460 (Micromeritics) at 77 K. The crystal morphologies of the samples were observed with a scanning electron microscope (SEM; ZEISS Ultra 55, Carl Zeiss) and transmission electron microscope (TEM, JEM-2100HR, JEOL) with a low loading energy and voltage. The thermogravimetric analysis (TGA) was performed on a TG analyzer (TG 209, Tarsus, NETZSCH) in a N_2_ atmosphere at a heating rate of 5 K min^−1^. Toluene and CH_4_ adsorption experiments were performed on an intelligent gravimetric analyzer (3H-2000PW) and RUBOTHERM magnetic suspension balance, respectively.

### Simulation method and parameters

**Fig. 1:**
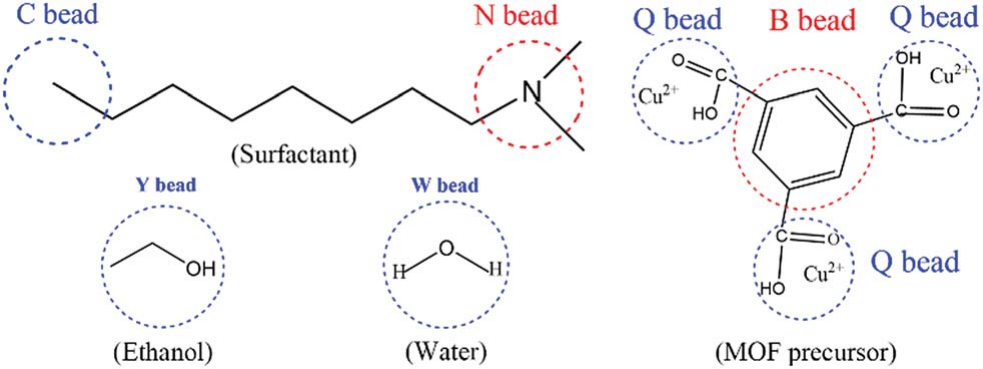
The coarse-grained models of the DMOA surfactant, ethanol, water, and MOF precursors (H_3_BTC and Cu^2+^) in the simulation system.

According to previous literature,^37,43^ a Gaussian chain of ‘‘springs and beads’’ is employed in the MesoDyn simulation method to represent the coarse-grained level. Typically, the springs can mimic the stretching behavior of a chain fragment, and the different species of beads stand for different monomers in the polymer molecules.^37^ Here, the coarse-grained model of the neutral DMOA surfactant could be deemed as a Gaussian chain with an N[C][C][C8] topology, where the C bead represents the hydrophobic alkyl chain and the N bead indicates the hydrophilic head, respectively. Moreover, the coarse-grained model of the ligand (H_3_BTC) could also be considered as a benzene ring (B bead) connected to three carboxyl groups.^44^ Q beads represented the carboxyl groups of H_3_BTC and Cu^2+^ (denoted as MOF precursors), while the Y and W beads stood for the solvent molecules of ethanol and water, respectively. The molecular structures and corresponding coarse-grained models of all components are illustrated in Fig. 1. In addition, the volume fractions of the DMOA surfactant, MOF precursors, E bead, and W bead were 1 : 1 : 2 : 2 in the present simulation system.

The interactions of various beads were represented as mean-field interaction energies (*υ*^−1^*ε*_*ij*_), which can be derived from the Flory–Huggins parameter *χ*_*ij*_ using eqn (1):^43^

1 *υ*^−1^*ε*_*ij*_ = *χ*_*ij*_*RT*

where *υ*^−1^*ε*_*ij*_ indicates the interaction energies between the pair of interacting bead types, the *χ*_*ij*_ parameter is taken from the blend simulation carried out for different beads, *R* refers to the molar gas constant, and *T* indicates the temperature (298 K). The Flory–Huggins interaction parameters *χij* between various beads used in simulation are summarized in Table S1.†

Similar to a previous simulation process,^39^ the dimensions of the simulated box were 32 nm × 32 nm × 32 nm. The bond length was set to 1.1543 to ensure isotropy of all grid-restricted operators.^43^ The bead diffusion coefficient was set as 10^−7^ cm^2^ s^−1^, the system's noise coefficient *Ω* = 75.019, the compressibility parameter was 10 kT, the simulated temperature was 298 K, the simulated time step was Δ*τ* = 50 ns, and the total simulation time was 1.0 ms (20 000 steps). In addition, in order to simulate the mixing environment during the synthetic process, a constant shear effect (*γ*) was introduced into the simulation system after 0.25 ms (5000 steps).^45^ The *X*-axis is the velocity direction, the *Y*-axis is the direction of the velocity gradient, and the *Z*-axis is the neutral axis.^39^ The program achieved a stable shear, that is, the velocity gradient was uniform: d*v*_*x*_/d*v*_*y*_ = *Û*_*γ*_ and the shear rate *γ* = 5 × 10^5^ s^−1^. All the simulations were carried out using the MesoDyn module of the commercial Materials Studio software package.

## Results and discussion

**Fig. 2:**
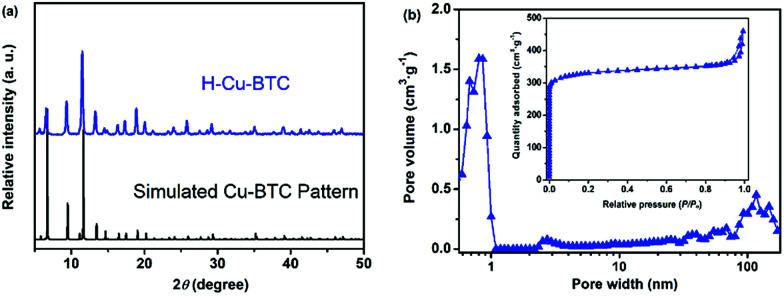
(a) Powder XRD patterns of H-Cu-BTC and the simulated Cu-BTC pattern; and (b) the N_2_ adsorption–desorption isotherms and corresponding pore size distributions of H-Cu-BTC.

To verify the crystalline structure of the hierarchically porous MOFs obtained from rapid synthesis, the powder XRD patterns of the as-synthesized H-Cu-BTC were compared to the simulated Cu-BTC patterns (CCDC: 112954).^46^ As shown in Fig. 2a, the XRD patterns of H-Cu-BTC exhibited sharp diffraction peaks, which were consistent with those of the simulated pattern, thereby confirming that the attained crystalline material is topologically identical to that of pristine Cu-BTC independent of the template.^47^ Moreover, the structure of H-Cu-BTC was examined by FTIR spectroscopy. As observed in Fig. S1a,† the FTIR spectra of the H-Cu-BTC clearly showed characteristic absorption bands at 1600, 1560, 1450, and 1380 cm^−1^, which were in excellent agreement with the previous reports of conventional Cu-BTC.^47,48^ However, the FTIR spectra of the H-Cu-BTC sample exhibited an absorption band in the narrow range of 1340–1210 cm^−1^ (C–N stretching vibration of surfactant DMOA) (Fig. S1b†), which could be attributed to the presence of some residual template. The mesostructure of the H-Cu-BTC sample was examined using the N_2_ adsorption–desorption isotherms and corresponding pore size distributions. As shown in Fig. 2b, a combination of type I and IV isotherms with an apparent hysteresis loop was observed for H-Cu-BTC, which suggested the co-existence of micropores and mesopores.^49^ Moreover, the pore size distribution curve of H-Cu-BTC, obtained from nonlocal density functional theory (NL-DFT) calculations, exhibited broad pore size distributions, ranging from the micro-, meso- to macroscale. However, conventional Cu-BTC (C-Cu-BTC) possesses only micropores with diameters of about 0.86 nm according to previous reports.^50^ In addition, H-Cu-BTC_1 and H-Cu-BTC_5 also exhibited mesoporous and macroporous structures, as observed in Fig. S2b.† More importantly, the shape and size of the hysteresis loops of hierarchically porous Cu-BTC depended on the amount of template (Fig. S2†), indicating that the porosity of hierarchically porous MOFs might be controlled by varying the concentration of the template. These results indicate the formation of mesopores and macropores in the H-MOF products. The corresponding textural properties of hierarchically porous MOFs are summarized in Table S2.† All of the as-synthesized hierarchically porous MOFs displayed lower Brunauer–Emmett–Teller (BET) surface areas than conventional Cu-BTC (1895 ± 84 m^2^ g^−1^),^41^ which can be attributed to the pore impenetration.^51,52^ However, a significantly higher mesopore volume (*V*_meso_) for hierarchically porous MOFs was observed than that of conventional Cu-BTC, further confirming the formation of mesostructures in the resulting hierarchically porous MOF materials.

**Fig. 3:**
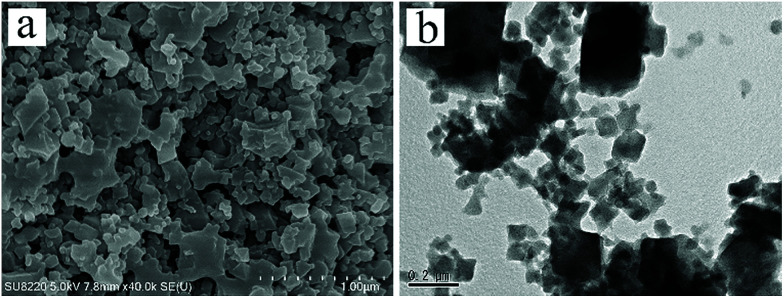
(a) SEM and (b) TEM images of the H-Cu-BTC sample.

The mesostructure of the corresponding H-Cu-BTC was further revealed by SEM and TEM. As observed in Fig. 3a, the SEM image showed that the H-Cu-BTC consisted of small and high- dispersive nanoparticles with diameters of about 500 nm and continuous pore voids were formed between these nanoparticles. The TEM image (Fig. 3b) further disclosed that abundant mesopores and macropores with pore sizes of 40–100 nm were formed in the final product, which is consistent with the N_2_ sorption results (Fig. 2b). Compared with conventional Cu-BTC with a crystal size of about 10 μm (Fig. S3†),^50^ the small nanocrystals in H-Cu-BTC can be attributed to the fact that the formed (Cu, Zn) hydroxy double salt (HDS) from ZnO particles has a high anion exchange rate, resulting in the rapid nucleation (10 min) between Cu^2+^ and BTC^3−^.^41,42^ The scan mapping clearly revealed the homogeneous distribution of elemental C, O, and Cu throughout the H-Cu-BTC crystals (Fig. S4†). In addition, the presence of traces of elemental N can be attributed to some templates residues in the H-Cu-BTC,^40^ which is consistent with the result of FTIR (Fig. S1b†). The thermal stability of the as-synthesized hierarchically porous MOFs was evaluated by TGA. As shown in Fig. S5,† the TGA curves indicate that the decomposition temperatures (∼330 °C) of hierarchically porous MOFs and conventional Cu-BTC are similar with the same thermal treatment, confirming that the introduction of mesopores and macropores into MOFs did not reduce their thermal stability.

**Fig. 4:**
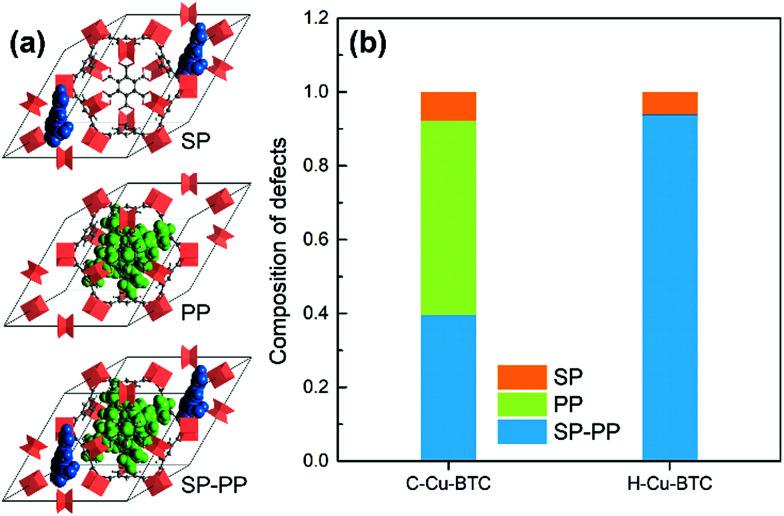
(a) Pore-blocking defects in Cu-BTC denoted as SP (side pocket) defects blocked by BTC, PP (primary pores) defects blocked by the template, and SP-PP defects; and (b) composition of the three defect types in C-Cu-BTC and H-Cu-BTC samples.

In addition, computational methods were used to evaluate the defects in hierarchical porous MOFs. According to previous literature,^53–55^ hierarchical porous Cu-BTC mainly contains pore blocking structure defects such as SP (side pocket) defects blocked by the ligand (BTC), PP (primary pore) defects blocked by the template, or SP-PP defects (combining both defects simultaneously), as presented in Fig. 4a. Since the side pockets of Cu-BTC are almost inaccessible for toluene molecules,^56,57^ the adsorption loading in the structures exclusively containing SP defects should be closest to the defect-free experimental results, and thus we assumed the SP defects as a “ideal” configuration in Cu-BTC when adsorbing toluene. A weighted summation algorithm was used to calculate the composition of each defect type in real materials, as shown in eqn (2):^58^2
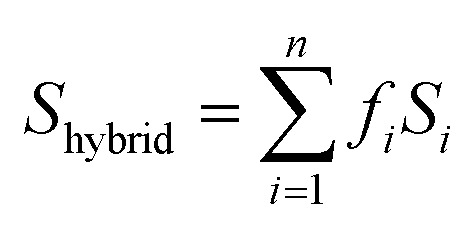
where *S*_hybrid_ refers to the BET surface area of the sample containing all possible structural defects; *S*_*i*_ represents the BET surface area of structures with SP defects (1884.72 m^2^ g^−1^), PP defects (1632.11 m^2^ g^−1^) and SP-PP defects (1059.17 m^2^ g^−1^), which was calculated using the RASPA package using helium (He) as the probe molecule;^59^*f*_*i*_ indicates the weight of composition of the corresponding structures. The value of *f*_*i*_ can be obtained by minimizing the error between *S*_hybrid_ and the BET surface area of conventional Cu-BTC (C-Cu-BTC) and hierarchical porous Cu-BTC with a patchwise model.^53^Fig. 4b shows the composition of structural defects of materials. As shown in Fig. 4b, the C-Cu-BTC contains 7.8% SP defects, 52.6% PP defects and 39.6% SP-PP defects, while the as-synthesized H-Cu-BTC product possesses 6.2% SP defects, negligible PP defects and 93.8% SP-PP defects. These analyses confirm that stable hierarchically porous Cu-BTC materials were successfully synthesized within 10 min under mild conditions by employing a new kind of DMOA surfactant as the template in a (Cu, Zn) HDS solution.

Order parameter *P* represents the mean-squared deviation from the homogeneity in the system and describes the influences of both phase separation and compressibility.^37^ The value of order parameter *P* was calculated using eqn (3):^60^3
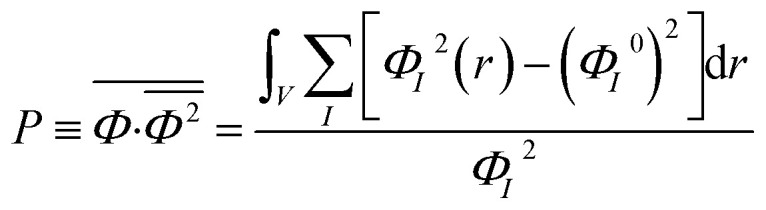
where *Φ* is the volume fraction of the surfactant and *V* is the volume of cells. The order parameter *P* and the free energy density of the system as a function of simulation time are presented in Fig. S6a.† As could be observed, the mesophase evolution of hierarchically porous MOFs could be divided into three stages. Specifically, in stage I, the order parameter *P* increased sharply, which marked the beginning of the formation of the ordered aggregation.^35^ In stage II, a steady shear *γ* (*γ* = 5 × 10^5^ s^−1^) was introduced into the system, which resulted in a marked decline in the order parameter *P* within a short time, indicating that the previous ordered system was broken.^37^ Afterwards, the order parameter *P* was increased rapidly. In stage III, the order parameter *P* remained relatively constant, indicating that the system had reached an equilibrium.^37^ In addition, the MesoDyn simulation could obtain the free energy change of the system, which could reflect the system stability.^35^ As shown in Fig. S6b,† firstly, the free energy of the system was decreased quickly (stage I), and then increased sharply in a split second when a steady shear *γ* (*γ* = 5 × 10^5^ s^−1^) was introduced (stage II). Finally, the free energy remained relatively stable, indicating that the system had reached a dynamic equilibrium.^61^

**Fig. 5:**
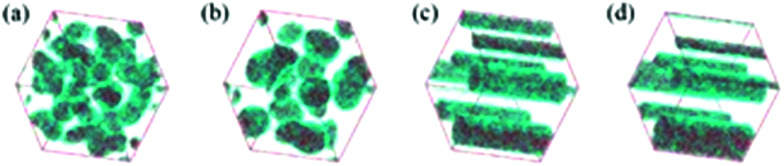
The aggregation evolution of DMOA surfactant supramolecular assembly at different simulation steps: (a) 1000 steps; (b) 5000 steps; (c) 10 000 steps; and (d) 20 000 steps.

Accordingly, Fig. 5 shows the snapshots of the aggregation evolution of the surfactant during hierarchically porous MOF formation. It can be seen from Fig. 5a that the synthesis system is homogeneous at the initial stage of simulation (1000 steps). Subsequently, with the extension in simulation time, the surfactant molecules gradually gather momentum and form aggregates through self-assembly (Fig. 5b), corresponding to stage I as observed in Fig. S6.† Furthermore, with the further extension of the synthesis time, the small aggregates were crashed while larger cylindroid micelles were generated (Fig. 5c), corresponding to stage II of Fig. S6.† This can be attributed to the different hydrophilic abilities between long-chain alkyl groups and the hydrophilic head of template molecules.^62^ Finally, cylindroid micelles were formed and the system reached a dynamic equilibrium (Fig. 5d), corresponding to stage III (Fig. S6†). The above analyses confirm that the introduction of surfactants into the synthesis system contributed to the formation of the supramolecular micelles.

**Fig. 6:**
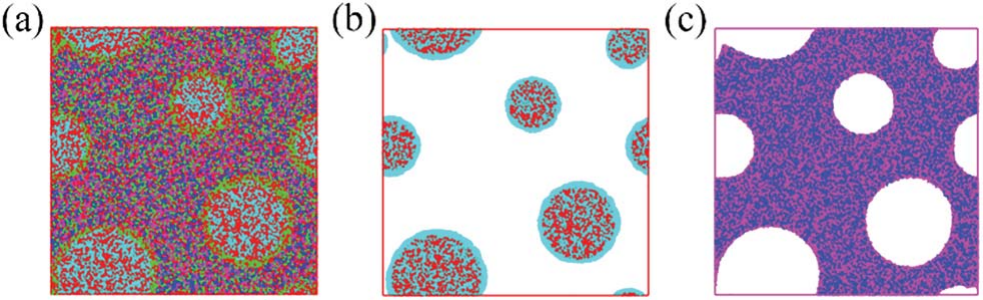
(a) Sectional view of the surfactant/MOF precursors/ethanol/water matrix, (b) surfactant, and (c) MOF precursors (red bead: C; turquoise bead: N; blue and crimson beads: MOF precursor). For simplification, ethanol and water molecules in (b and c) are omitted.

**Scheme 1:**
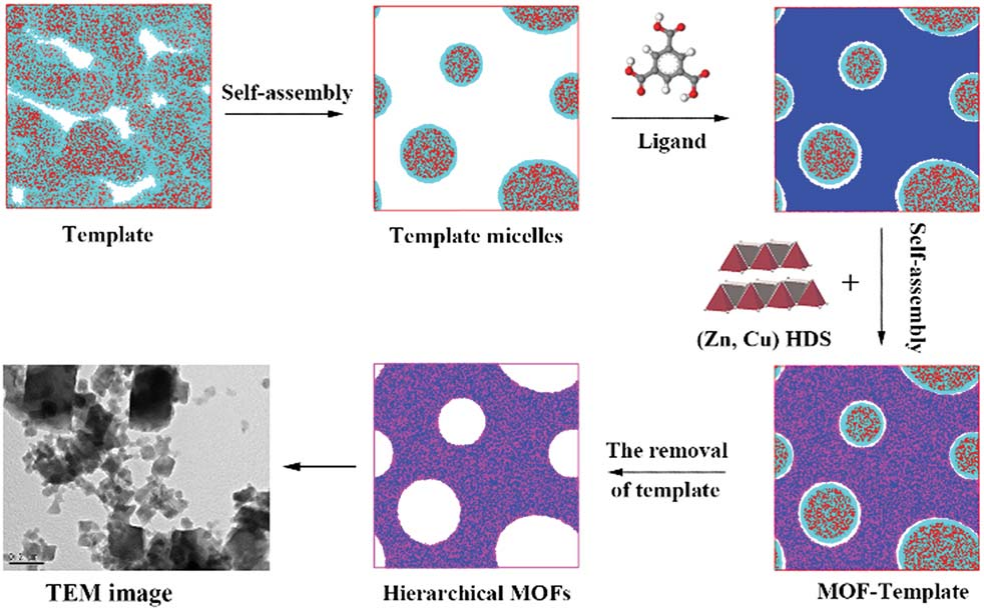
Schematic representation of the hierarchically porous MOF synthesis by using a template in a (Cu, Zn) hydroxy double salt (HDS) solution.

To more clearly observe the structure of the surfactant micelles (Fig. 6a), a representative sectional view of the cylindroid micelles was carefully examined. As shown in Fig. 6b, the surfactant molecules aggregated in the form of cylindroid micelles, in which the cylindroid micelles were comprised of a hydrophobic inner core (N bead) and a hydrophilic shell (C bead). In addition, the MOF precursors gradually aggregated on the outer surface of template micelles (Fig. 6c), which could be attributed to the appropriate electrostatic interactions between the MOF precursors and the micelles.^43,63^ Afterwards, larger pores were formed after the removal of template micelles by simple activation, which were located at the positions of template micelles.^64^ Such results supported the previous hypothesis by the Qiu group suggesting that hierarchically porous MOFs could be self-assembled from MOF precursors in the presence of surfactant micelles as the supramolecular templates at the molecular level.^64^ Based on the above analysis and literature precedents, a schematic representation of the cooperative template method preparation of hierarchically porous MOFs is illustrated in Scheme 1. In the initial stages, the introduced ZnO particles react with Cu(NO_3_)_2_ to form (Zn, Cu) HDS, which has a high anion exchange rate in the ligand solution. This is a key step to rapidly form H-MOF crystals.^41,42,65^ Meanwhile, the introduced DMOA surfactant forms supramolecular micelles through self-assembly, a key step to generate mesopores and macropores.^64^ Upon the addition of (Cu, Zn) HDS to the micelle solution, the Cu^2+^ and BTC^3−^ rapidly self-assemble on the surface of the template micelles due to the high anion exchange rate.^41,64^ As a result, mesopores and macropores are produced in the network after the removal of the template micelles through activation and drying.^64,66^ The obtained experimental data are consistent with the simulation results and the proposed synthetic mechanism, as confirmed by SEM and TEM observations (Fig. 3).

**Fig. 7:**
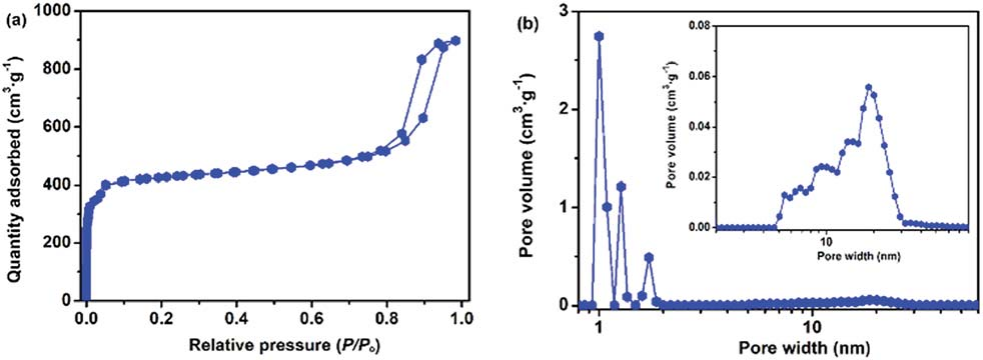
(a) The N_2_ adsorption–desorption isotherms and (b) corresponding pore size distributions of H-ZIF-8.

In addition to the hierarchically porous Cu-BTC, other hierarchically porous MOFs such as hierarchically porous ZIF-8 (denoted as H-ZIF-8, ESI) could also be synthesized by employing the DMOA surfactant as the template. The XRD patterns of the attained H-ZIF-8 agree well with those of the simulated ZIF-8 (Fig. S7†), thereby confirming that the obtained product possesses a representative ZIF-8 framework.^67^ As shown in Fig. 7a, the N_2_ adsorption–desorption curves of the attained H-ZIF-8 display a typical type-IV isotherm, corresponding to a hybrid meso- and microporous material. Furthermore, the pore size distributions of H-ZIF-8 indicate that the product is a hybrid micro-, meso-, and macroporous material (Fig. 7b). Moreover, SEM and TEM images indicate that the coexistence of meso- and macropores in the resulting H-ZIF-8 (Fig. S8†). Interestingly, other surfactants such as *N*,*N*-dimethyldodecylamine (DMDA) could also be used as templates for the room-temperature rapid synthesis of stable hierarchically porous MOFs (denoted as H-Cu-BTC_A, see Fig. S9–S12† for details), confirming the feasibility and universality of our rapid synthetic mechanism. More importantly, the pore size distributions of the hierarchically porous MOFs varied based on the type of surfactant (Fig. S13†), thereby allowing facile tailoring of the porosity properties. In addition, hierarchically porous ZIF-8 (denoted as H-ZIF-8_A, ESI) could also be synthesized using DMDA as the template (see Fig. S14 and 15 for details†). Furthermore, the TGA result indicates that the as-synthesized H-ZIF-8 and H-ZIF-8_A products have excellent thermal stability (Fig. S16†). Moreover, the porosity of hierarchically porous ZIF-8 depends on the amount and type of surfactant, as confirmed by Table S2† and Fig. S17 and 18,† respectively. All these remarks imply that our rapid synthesis strategy is versatile.

**Fig. 8:**
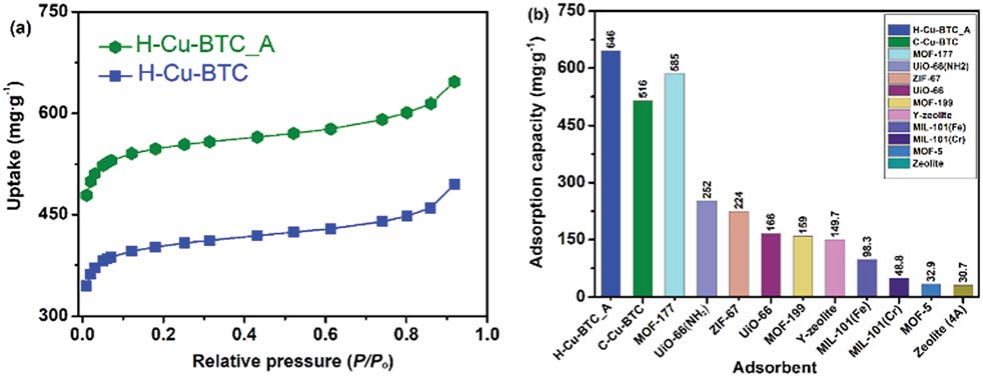
(a) Toluene sorption isotherms at 298 K on H-Cu-BTC_A and H-Cu-BTC samples, and (b) the saturation adsorption capacities of toluene on various adsorbents at 298 K.

Toluene, one of the most common volatile organic compounds (VOCs), is a notorious chemical pollutant due to its malodor, carcinogen, toxicity, and high penetration rate even at a very low concentration.^68,69^ Toluene has been widely adopted in various industrial processes, and has thus caused serious air pollution and social health hazard effects.^70,71^ Accordingly, MOFs, one of most attractive adsorbents, exhibited great potential for capturing and removing toluene with low concentrations (<1000 ppm_v_).^71–73^Fig. 8a shows the toluene sorption isotherms on various hierarchically porous MOFs (H-Cu-BTC_A and H-Cu-BTC) at 298 K. It can be seen that the toluene adsorption isotherm on H-Cu-BTC_A can be divided into three stages. At low relative pressures (*P*/*P*_o_ < 0.01), the isotherm exhibited a steep rise in the uptake followed by a plateau, corresponding to the monolayer adsorption of toluene in micropores.^72^ With the increase in relative pressures (*P*/*P*_o_ > 0.60), the isotherm still showed an increase in the adsorbed amount of toluene, which is related to the capillary condensation of toluene in mesopores and macropores.^74^ Similar profiles are also observed for toluene adsorption on the H-Cu-BTC sample. Comparatively, the loading of toluene on H-Cu-BTC_A is always higher than that on H-Cu-BTC due to the fact that more mesopores are present in H-Cu-BTC_A than H-Cu-BTC (Table S2†), thus allowing them to freely and rapidly diffuse into the internal active sites.^21^ Specifically, the saturation adsorption capacity of toluene on H-Cu-BTC_A is 646 mg g^−1^, which is 25% higher than the reported value of conventional Cu-BTC (516 mg g^−1^),^75^ and also greatly surpasses those of other conventional MOFs and zeolites (Fig. 8b).^74,76–78^ The as-synthesized H-ZIF-8 also shows remarkably higher toluene adsorption capacity (963 mg g^−1^) than conventional porous materials (Fig. S19†). In addition, H-Cu-BTC_A exhibits the maximum uptake of CH_4_ is 112.4 mg g^−1^ at 3 MPa (Fig. S20†), which is higher than those of porous materials such as zeolites.^79^ Moreover, the as-prepared hierarchically porous MOF materials including Cu-BTC and ZIF-8 have great potential for the capture of dyes such as Congo red (CR, see Fig. S21 and 22 for details†). These results indicate that H-MOFs are promising candidates for VOC adsorption, dye degradation, and energy gas storage.

## Conclusions

In summary, two stable hierarchically porous metal–organic frameworks named Cu-BTC and ZIF-8 were successfully synthesized by using a new surfactant as the template under mild conditions. Structural analysis revealed that the as-prepared products had multimodal hierarchically porous structures with macro-, meso-, and micropores while retaining the parent topology. Furthermore, the synthetic strategy is highly versatile, which allows easy tuning of the porosity properties of the hierarchically porous MOFs by controlling the amount and type of surfactant employed. The results of mesodynamics (MesoDyn) simulation demonstrate that the introduced surfactant (*N*,*N*-dimethyloctylamine) led to the formation of supramolecular micelles, and then the MOF precursors were self-assembled on the surface of the template micelles, which accounts for the synthetic mechanism of hierarchically porous MOFs at the molecular level. In addition, the as-synthesized hierarchically porous Cu-BTC exhibited a maximum toluene adsorption capacity of 646 mg g^−1^ at 298 K, which is a 25% increase compared with that of pristine Cu-BTC (516 mg g^−1^) and is much higher than other microporous MOFs and zeolites. The synthetic strategy and mechanism explored in this work are of great importance to the large-scale industrial fabrication of various hierarchically porous MOFs, which is advantageous for their potential applications in various fields involving both large and small guest molecules, such as adsorption, separation, drug delivery, *etc.*

## Conflicts of interest

There are no conflicts to declare.

## Supplementary Material

NA-001-C8NA00262B-s001
