# Supplementary material for: Templated fabrication of hierarchically porous metal–organic frameworks and simulation of crystal growth
Source: Nanoscale Adv. 2018 Dec 12;1(3):1062–9. doi: 10.1039/c8na00262b (PMC9473183; doi:10.1039/c8na00262b)
Supplement: NA-001-C8NA00262B-s001 [file NA-001-C8NA00262B-s001.pdf]

Electronic Supplementary Material (ESI) for Nanoscale Advances.
This journal is © The Royal Society of Chemistry 2018

Electronic Supplementary Information (ESI)

Templating fabrication of hierarchically porous metal–organic frameworks and simulation of crystal growth

Chongxiong Duan,[a] Hang Zhang,[a] Minhui Yang,[a] Feier Li,[a] Yi Yu,[a] Jing Xiao,[a] Hongxia Xi*,[a, b]
[a]School of Chemistry and Chemical Engineering, South China University of Technology, Guangzhou 510640, China.
[b]Guangdong Provincial Key Laboratory of Atmospheric Environment and Pollution Control, South China University of Technology, Guangzhou Higher Education Mega Centre, Guangzhou 510006, PR China.

Experimental section

Chemicals. Copper nitrate trihydrate ($Cu(NO_3)_2 \cdot 3H_2O$), 1,3,5-benzenetricarboxylic acid (H_3BTC), zinc acetate dihydrate ($Zn(CH_3CO_2)_2 \cdot 2H_2O$), 2-methylimidazole (2Im), zinc oxide (ZnO), *N*,*N*-dimethyloctylamine (DMOA), *N*,*N*-dimethyldodecylamine (DMDA), and *N,N*-dimethylformamide (DMF), above chemicals were purchased from J&K or aladdin Chemical Ltd, and utilized without further purification.

Solvothermal synthesis of conventional Cu-BTC

The conventional microporous Cu-BTC was prepared according to the procedures reported,[1] and the obtained product is denoted as C-Cu-BTC.

Rapid room-temperature synthesis of hierarchically porous Cu-BTC with *N*,*N*-dimethyldodecylamine as template

The experimental procedures are similar to the synthesis of H-Cu-BTC, except the *N*,*N*-dimethyloctylamine (DMOA) was replaced by *N*,*N*-dimethyldodecylamine (DMDA). The resulting product is denoted as H-Cu-BTC_A.

Rapid synthesis of hierarchically porous ZIF-8 under facile conditions

The experimental procedures are similar to the previously reported methods,[2, 3] firstly, 5 mmol of zinc oxide (ZnO) was added to 10 mL of deionized water as solution A, and 5 mmol of zinc acetate dihydrate ($Zn(CH_3CO_2)_2 \cdot 2H_2O$) was added to 5 mL of *N*,*N*-dimethylformamide (DMF) as solution B. After that, two solutions were mixed under fast magnetic stirring (denoted as solution C), and continue stirring for 16 h. The formation of gel-like viscous fluid indicates that the

formation of (Zn, Zn) hydroxy double salt (HDS).[3] After that, 3 mmol of 2-methylimidazole (2Im) and 3 mmol of DMOA were added to 15 mL methanol (denoted as solution D), and stirring for 15 min. Then, 2 mL of (Zn, Zn) HDS suspension was added to solution D, and then continue stirring for 10 min. Subsequently, the precipitate was collected by filtered and immersed in ethanol four times at 60 °C for 48 h, and then dried overnight in an oven at 120 °C. The resulting product is denoted as H-ZIF-8. Similarly, hierarchically porous ZIF-8 synthesized with DMDA as template is denoted as H-ZIF-8_A.

Table S1 Flory-Huggins interaction parameter χ_{ij} between various beads used in this work.

Bead ^{a}	Y	W	B	Q	N	C
Y	0.00	0.196	0.514	4.08	0.156	3.53
W		0.00	2.74	4.50	-0.498	7.28
B			0.00	4.18	2.29	1.43
Q				0.00	4.23	13.9
N					0.00	3.08
C						0.00

Table S2 Textural properties of hierarchically porous Cu-BTC and conventional Cu-BTC.

Sample	$S_{BET}{}^{a}$ [m^{2}·g^{-1}]	$S_{micro}{}^{b}$ [m^{2}·g^{-1}]	$S_{meso}{}^{c}$	$V_{t}{}^{d}$ [cm^{3}·g^{-1}]	$V_{meso}{}^{e}$ [cm^{3}·g^{-1}]	$V_{micro}{}^{f}$ [cm^{3}·g^{-1}]
H-Cu-BTC	1110	926	184	0.61	0.17	0.43
H-Cu-BTC_1	1347	1153	194	0.66	0.12	0.54
H-Cu-BTC_5	765	640	125	0.44	0.14	0.30
H-Cu-BTC_A	563	453	110	0.59	0.38	0.21
H-ZIF-8	1660	1456	204	1.35	0.78	0.57
H-ZIF-8_1	1652	1469	183	1.24	0.70	0.54
H-ZIF-8_5	1617	1378	239	1.27	0.74	0.53
H-ZIF-8_A	1350	1098	252	1.19	0.68	0.51

$^{a}S_{BET}$: Brunauer–Emmett–Teller (BET) surface area; $^{b}S_{micro}$: micropore surface area; $^{c}S_{meso}$: mesopore surface area; $^{d}V_{t}$: total pore volume; $^{e}V_{meso}$: mesopore volume; $^{f}V_{micro}$: micropore volume.

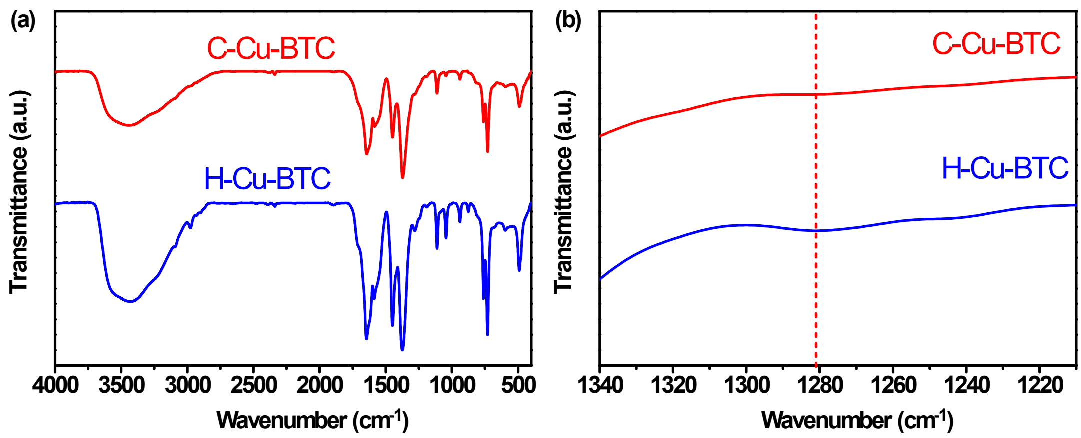

Fig. S1 (a) FTIR spectra of H-Cu-BTC and conventional Cu-BTC (C-Cu-BTC) samples, and (b) FTIR spectra of H-Cu-BTC sample in the narrow region of 1340–1210 cm^{-1}.

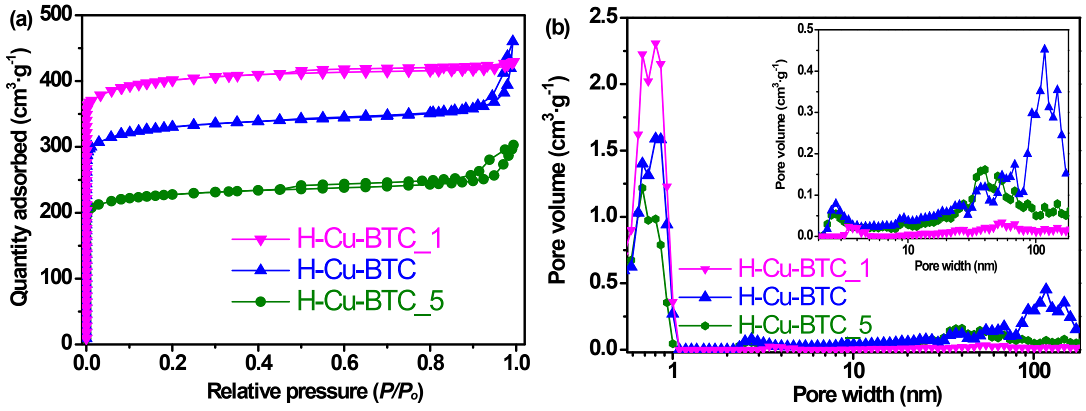

Fig. S2 (a) N_2 adsorption–desorption isotherms and (b) corresponding pore size distributions of H-Cu-BTC, H-Cu-BTC_1, and H-Cu-BTC_5 samples.

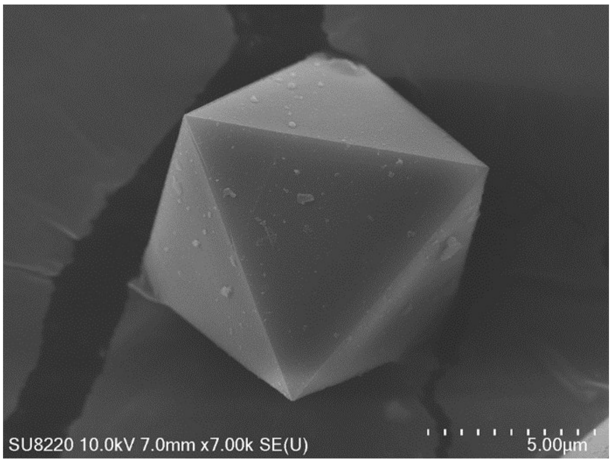

Fig. S3 SEM image of conventional Cu-BTC sample.

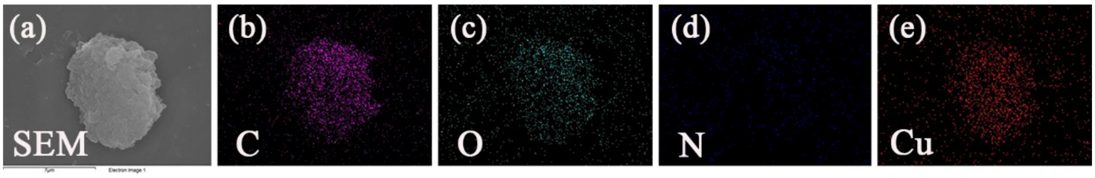

Fig. S4 Elemental distribution maps of H-Cu-BTC: (a) SEM, (b) C, (c) O, (d) N, and (e) Cu.

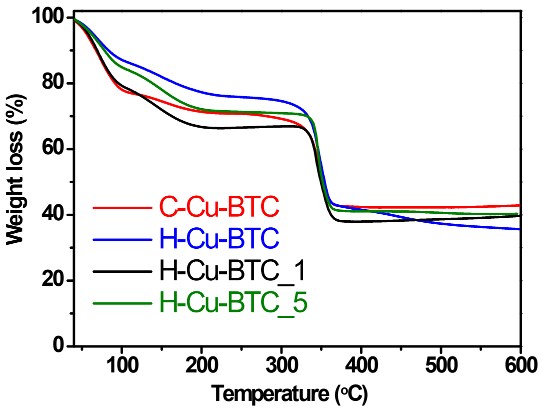

Fig. S5 Thermogravimetric analysis (TGA) of conventional Cu-BTC (C-Cu-BTC) and H-MOFs.

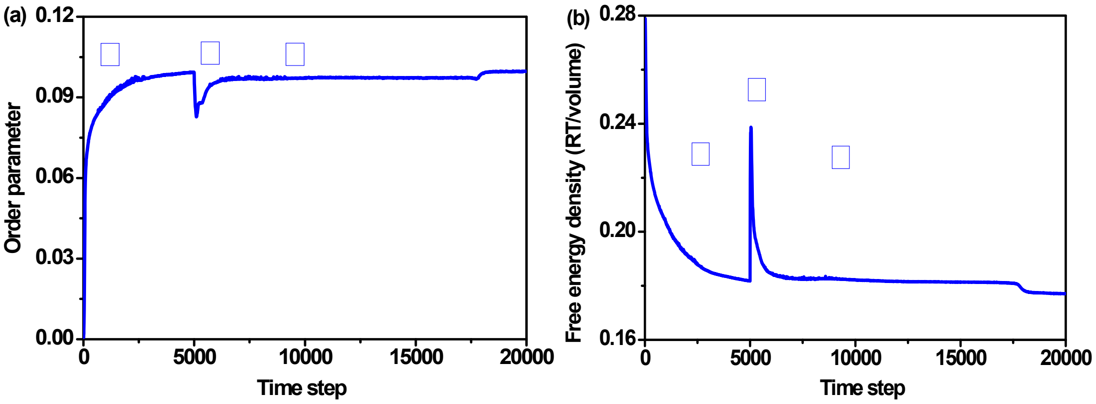

Fig. S6 (a) Time evolution of order parameter P, and (b) the free energy density plot with time step during the mesophase formation of hierarchically porous MOFs.

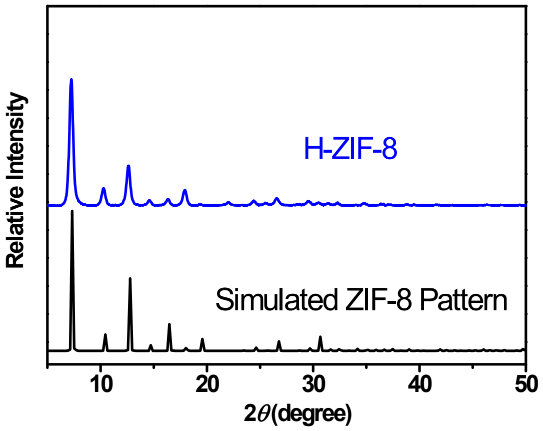

Fig. S7 Powder XRD patterns of H-ZIF-8 and the simulated ZIF-8 pattern.

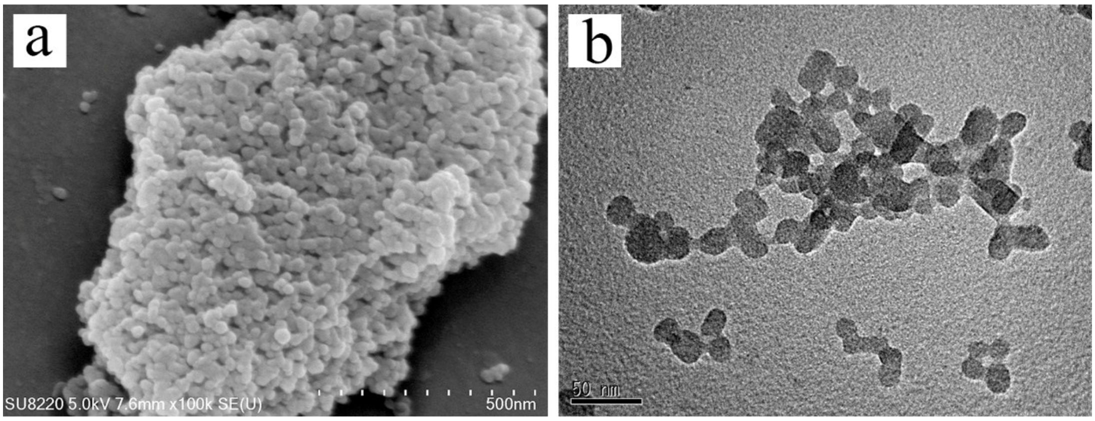

Fig. S8 (a) SEM and (b) TEM images of H-ZIF-8 sample.

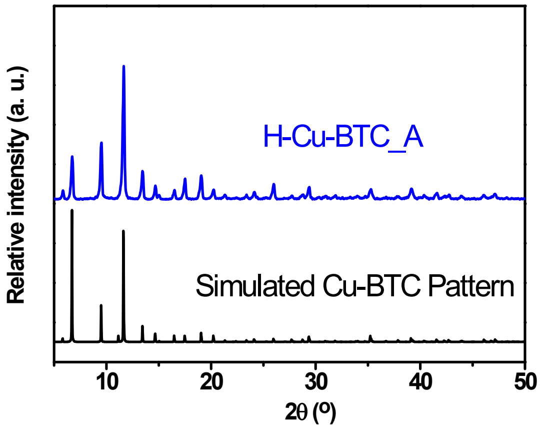

Fig. S9 Powder XRD patterns of H-Cu-BTC_A and the simulated Cu-BTC pattern.

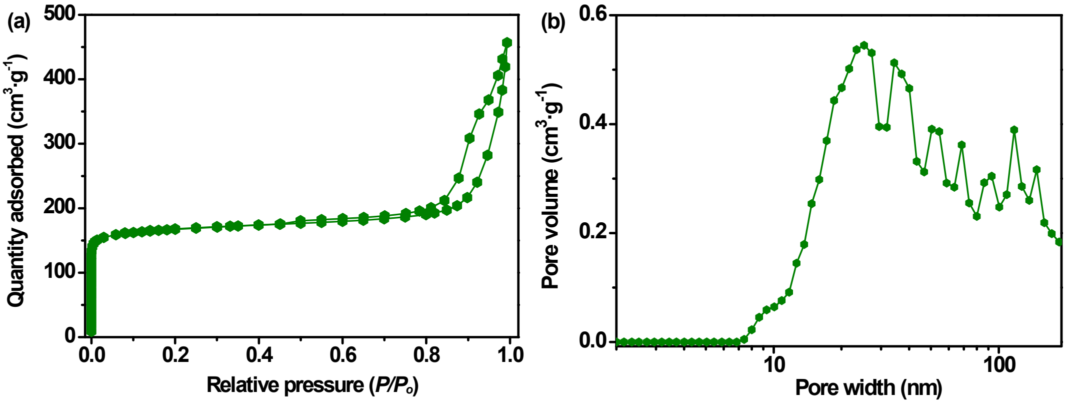

Fig. S10 (a) N_2 adsorption–desorption isotherms and (b) pore size distributions of H-Cu-BTC_A.

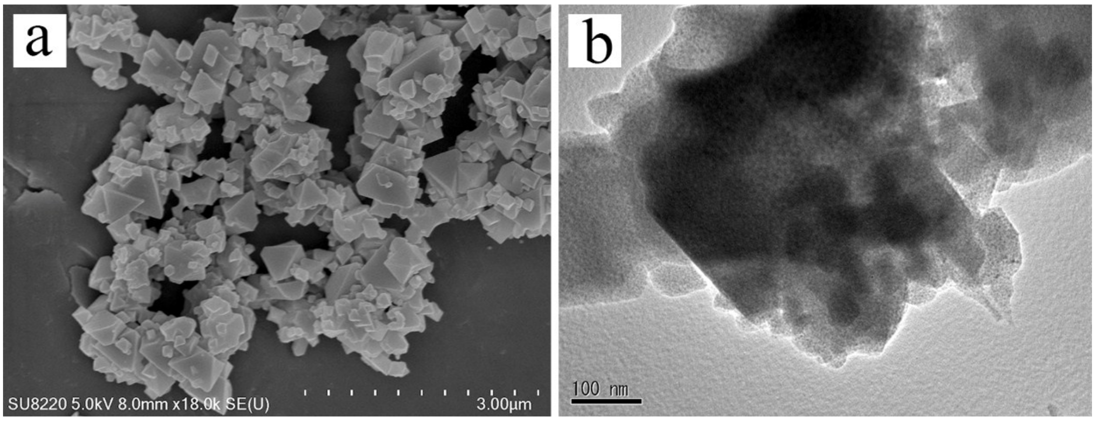

Fig. S11 (a) SEM and (b) TEM images of H-Cu-BTC_A sample.

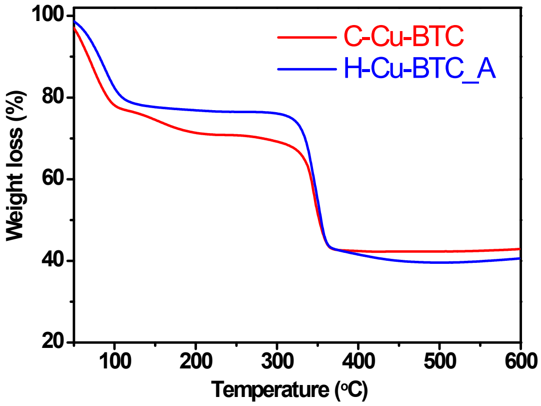

Fig. S12 TGA of H-Cu-BTC-A and C-Cu-BTC samples.

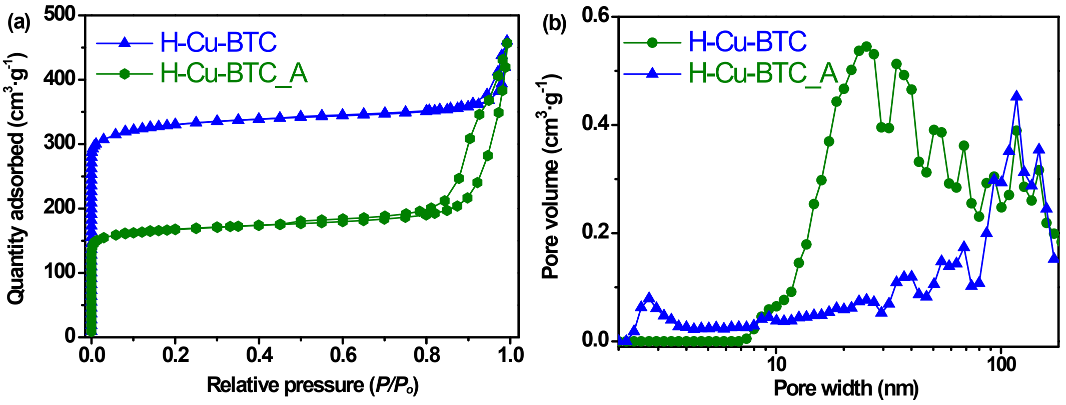

Fig. S13 (a) The N_2 adsorption–desorption isotherms and (b) the corresponding pore size distributions of H-Cu-BTC and H-Cu-BTC_A samples.

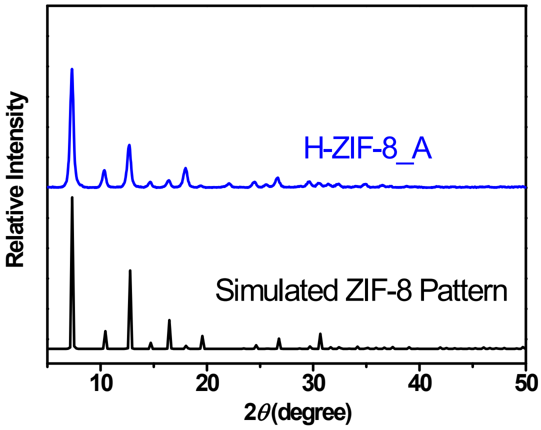

Fig. S14 Powder XRD patterns of H-ZIF-8_A and the simulated ZIF-8 pattern.

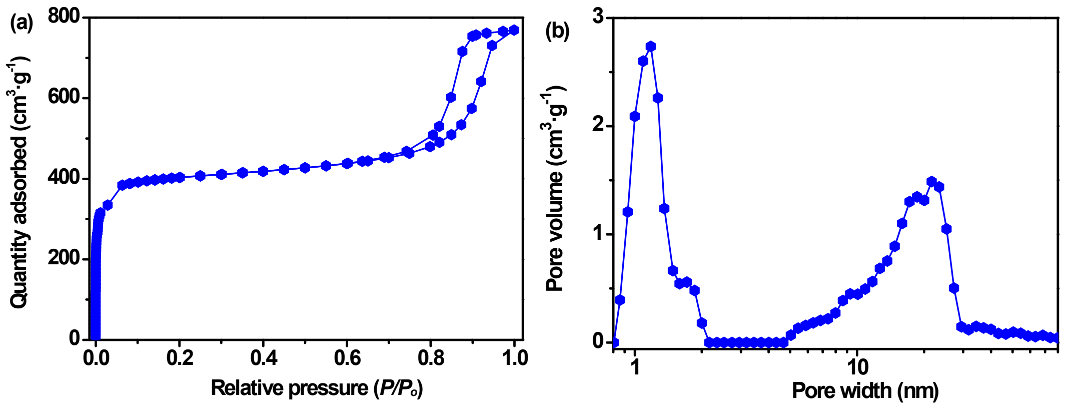

Fig. S15 (a) N_2 adsorption–desorption isotherms and (b) pore size distributions of H-ZIF-8_A.

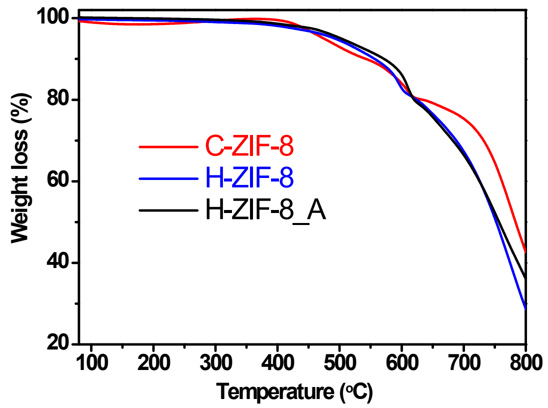

Fig. S16 TGA of H-ZIF-8, H-ZIF-8_A, and conventional ZIF-8 (C-ZIF-8) samples.

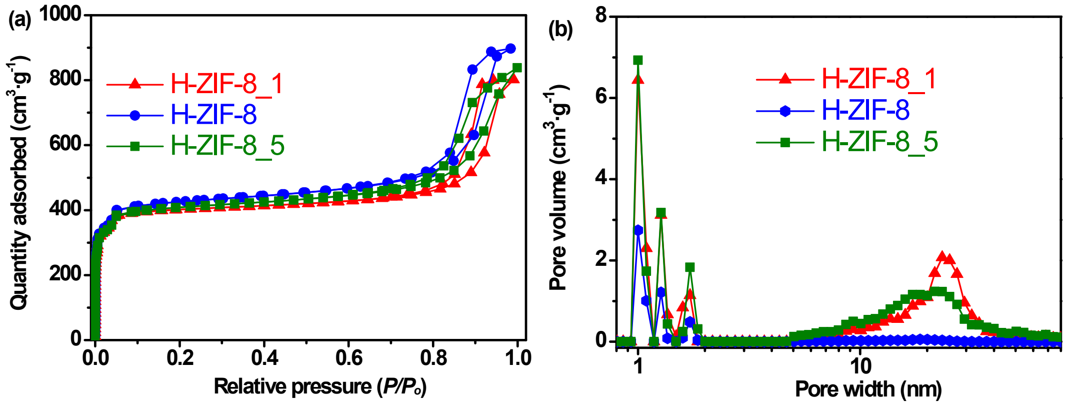

Fig. S17 (a) N_2 adsorption–desorption isotherms and (b) corresponding pore size distributions of H-ZIF-8_1, H-ZIF-8, and H-ZIF-8_5 samples.

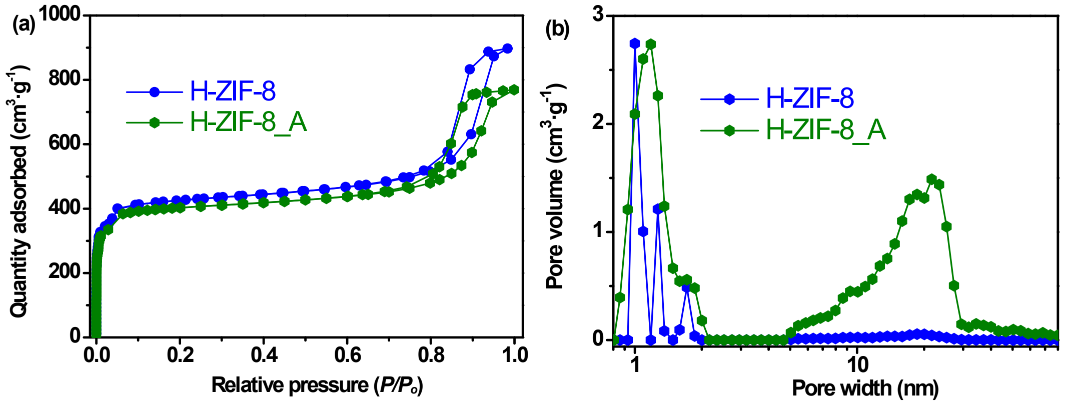

Fig. S18 (a) N_2 adsorption–desorption isotherms and (b) corresponding pore size distributions of H-ZIF-8 and H-ZIF-8_A samples.

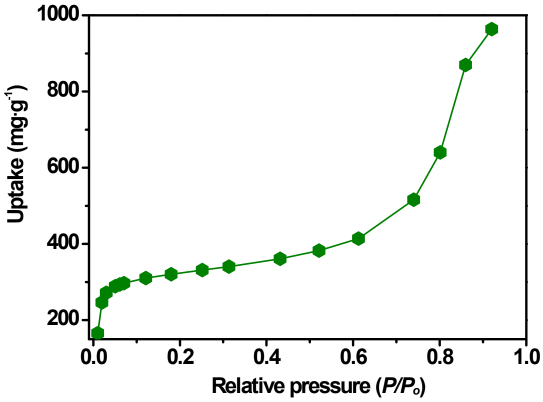

Fig. S19 Gaseous toluene sorption isotherms at 298K on H-ZIF-8 sample.

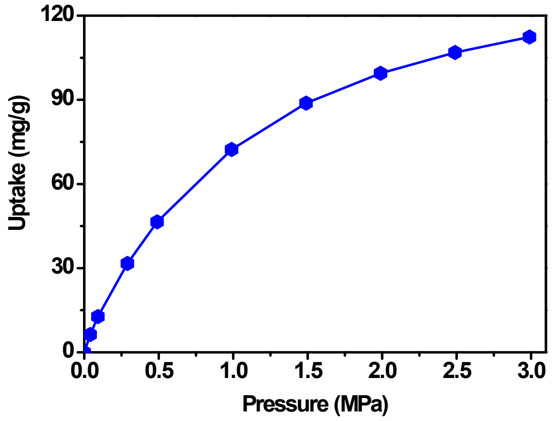

Fig. S20 CH_4 adsorption curve of H-Cu-BTC_A sample at 298 K.

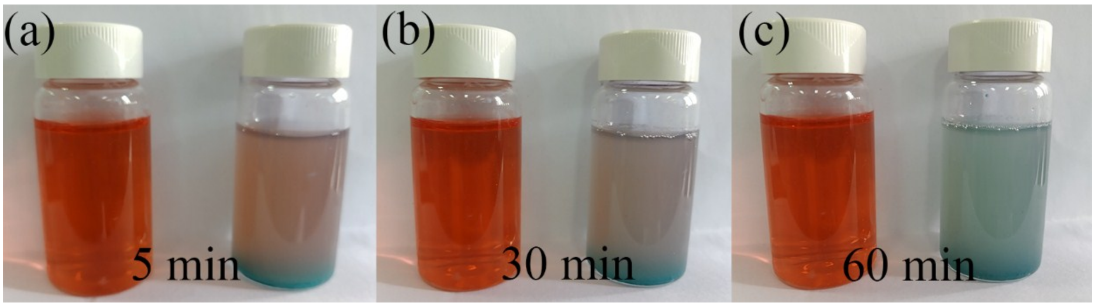

Fig. S21 Photograph of decoloration process for congo red (CR, $C_0 = 30$ mg·L^{-1}) solution with H-Cu-BTC_A (m = 20 mg) adsorbent (right) at different times: (a) 5 min; (b) 30 min; and (c) 60 min.

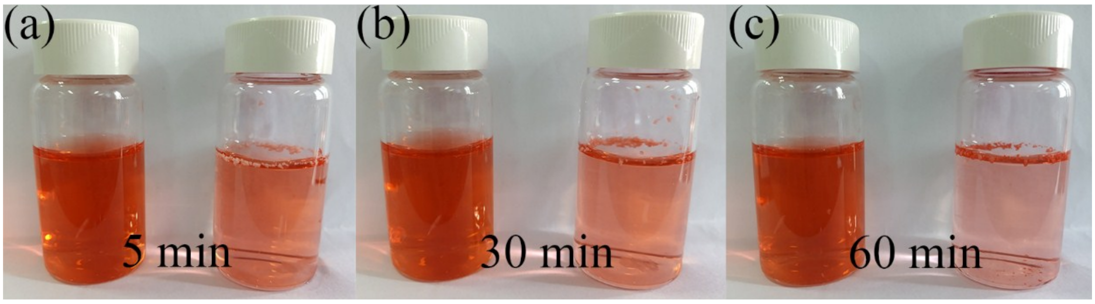

Fig. S22 Photograph of decoloration process for CR ($C_0 = 30$ mg·L^{-1}) solution with H-ZIF-8 (m =

20 mg) adsorbent (right) at different times: (a) 5 min; (b) 30 min; and (c) 60 min.

References

1. C. Duan, F. Li, H. Zhang, J. Li, X. Wang and H. Xi, *RSC Adv.*, 2017, 7, 52245-52251.
2. C. Duan, F. Li, L. Li, H. Zhang, X. Wang, J. Xiao and H. Xi, *CrystEngComm*, 2018, 20, 1057-1064.
3. J. Zhao, W. T. Nunn, P. C. Lemaire, Y. Lin, M. D. Dickey, C. J. Oldham, H. J. Walls, G. W. Peterson, M. D. Losego and G. N. Parsons, *J. Am. Chem. Soc.*, 2015, 137, 13756-13759.
